# Impact of host genetics on gut microbiome: Take‐home lessons from human and mouse studies

**DOI:** 10.1002/ame2.12134

**Published:** 2020-09-17

**Authors:** Inbal Cahana, Fuad A. Iraqi

**Affiliations:** ^1^ Department of Human Microbiology and Immunology Sackler Faculty of Medicine Tel‐Aviv University Tel‐Aviv Israel

**Keywords:** host genetic background, intestinal microbiome in human and mouse, microbial variations and profiles, take‐home lessons

## Abstract

The intestinal microbiome has emerged as an important component involved in various diseases. Therefore, the interest in understanding the factors shaping its composition is growing. The gut microbiome, often defined as a complex trait, contains diverse components and its properties are determined by a combination of external and internal effects. Although much effort has been invested so far, it is still difficult to evaluate the extent to which human genetics shape the composition of the gut microbiota. However, in mouse studies, where the environmental factors are better controlled, the effect of the genetic background was significant. The purpose of this paper is to provide a current assessment of the role of human host genetics in shaping the gut microbiome composition. Despite the inconsistency of the reported results, it can be estimated that the genetic factor affects a portion of the microbiome. However, this effect is currently lower than the initial estimates, and it is difficult to separate the genetic influence from the environmental effect. Additionally, despite the differences between the microbial composition of humans and mice, results from mouse models can strengthen our knowledge of host genetics underlying the human gut microbial variation.

## Graphical abstract

The gut microbiome, often defined as a complex trait, contains diverse components and its properties are determined by a combination of environmental and genetic factors. It is still difficult to evaluate, which human genetics shape the composition of the gut microbiota, however mouse models were proven to be a powerful tool for this. The purpose of this paper is to provide a current assessment of the role of human and mouse host genetics in shaping the gut microbiome composition.

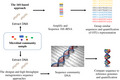

## 1 HUMAN GUT MICROBIOME

The human body is constantly interacting with various microorganisms that cover its external surfaces and are collectively called the microbiome. The gut microbiome, which contains more than 1000 species of bacteria, is considered the largest and most studied in the field to date.^1^, ^2^ Although it was previously thought that the gut microbiome formation begins at birth, recent studies suggest that this initial development occurs at prenatal stages.^3^ After birth, especially during the first 3 years of life, the development of this microbiome is accelerated mainly due to environmental exposure. It has been demonstrated that factors, such as nutrition,^4^ antibiotics,^5^ mode of delivery, and cessation of breast‐feeding,^6^ have a major impact on its shape in adults.^7^ In addition to the dynamics and variability of the microbial communities within the individual, the composition of the microbiome varies significantly between individuals; for example, the species that inhabit the gut, the relative ratios among different bacteria, and the identity of the dominant species.^8^, ^9^, ^10^

In the past decade, especially after the establishment of the Human Microbiome Project,^10^ great progress has been made in understanding the importance of the gut microbiome in maintaining our health.^11^, ^12^ It has been discovered that changes in the gut microbiome are associated with various diseases and health conditions.^13^ Increasing evidence of intestinal microbiota involvement in pathogenesis and disease development reveals its potential as a promising therapeutic target for disease management, prevention, and cure.^14^ Thus, considerable effort is focused on understanding the factors that shape microbial composition. The gut microbiome is a complex trait affected by multiple factors, including genetics and environment.^15^ The typical methods for characterizing the microbiome are either with 16S ribosomal RNA (rRNA) gene sequencing, shotgun, or high‐throughput metagenome sequencing techniques.^8^ These techniques allow quantification of taxa or gene functions, which can be used as a database for different ecological metrics that characterize diversity in a sample or within a population.^15^ The early studies focused on 16S rRNA sequences which are relatively short, often conserved within a species, and generally different between species. Many 16S rRNA sequences have been found, which do not belong to any known cultured species, indicating that there are numerous non‐isolated organisms. 16S rRNA sequencing has been developed in response to the need for more rapid and accurate identification of complex microbiome.^16^ The 16S rRNA gene “Marker gene”^17^ codes for a ribosomal subunit that is widely conserved among bacteria and contains hypervariable regions V1‐V9 interspersed among conserved regions of its sequence. These hypervariable regions are unique to each bacterial species, allowing for classification or taxonomy. The conserved regions, on the other hand, allow for the development of universal primers that bind to known sequences shared among most bacteria. The 16S rRNA gene is amplified from total extracted DNA using universal primers to target the conserved regions of the gene, and the resulting PCR products are sequenced to identify the bacterial species present.^18^ For the nine hypervariable regions, some regions better characterize bacteria and the choice of which region, and therefore, the appropriate primers to use are an important step in study design.^19^ Regardless of the sequencing method, final results are represented in operational taxonomic units (OTUs), which is a sequence identifying an organism usually at the genus or species level. OTUs are based on similarity, in which, the similarity between a pair of sequences is computed as the percentage of sites that agree in a pairwise sequence alignment. A common similarity threshold used is 97%, which was derived from an empirical study that showed most strains had 97% 16S rRNA sequence similarity.^20^

While with the advancement in refinements of DNA amplification, the proliferation of computational power and bioinformatics tools have greatly aided the analysis of DNA sequences recovered from environmental samples, allowing the adaptation of shotgun sequencing to metagenomic samples (known also as whole metagenome shotgun or WMGS sequencing). Shotgun sequencing is a method used for sequencing random DNA strands.^21^, ^22^ The approach, used to sequence many cultured microorganisms and the human genome, randomly shears DNA, sequences many short sequences (reads), overlapping ends of different reads to assemble them into a continuous sequence and subsequently reconstruct them into a publically consensus sequence, using sophisticated computer programs. Shotgun metagenomics provides information both about, which organisms are present and what metabolic processes are possible in the community.^23^ Because the collection of DNA from an environment is largely uncontrolled, the most abundant organisms in an environmental sample are most highly represented in the resulting sequence data. To achieve the high coverage needed to fully resolve the genomes of under‐represented community members, large samples, often prohibitively so, are needed. In contrast, the random nature of shotgun sequencing ensures that many of these organisms, which would otherwise go unnoticed using traditional culturing techniques, will be represented by at least some small sequence segments.^24^

Finally, recently a high‐throughput sequencing technique, which does not require cloning the DNA before sequencing, removing one of the main biases and bottlenecks in environmental sampling was developed. The first metagenomic studies conducted using high‐throughput sequencing used massively parallel 454 pyrosequencing.^25^ Three other technologies commonly applied to environmental sampling are the Ilumina MiSeq and HiSeq, and the Applied Biosystems SOLid systems.^26^ These techniques for sequencing DNA generate shorter fragments than Sanger sequencing; 454 pyrosequencing typically produces ~400 bp reads, Illumina MiSeq produces 400‐700 bp reads (depending on whether paired‐end options are used), and SOLiD produce 25‐75 bp reads.^27^ Using this technique, it is possible to comprehensively sequence the entire microbiota in a given biological sample and subsequently compare it to a publically consensus sequence.

**FIGURE 1:**
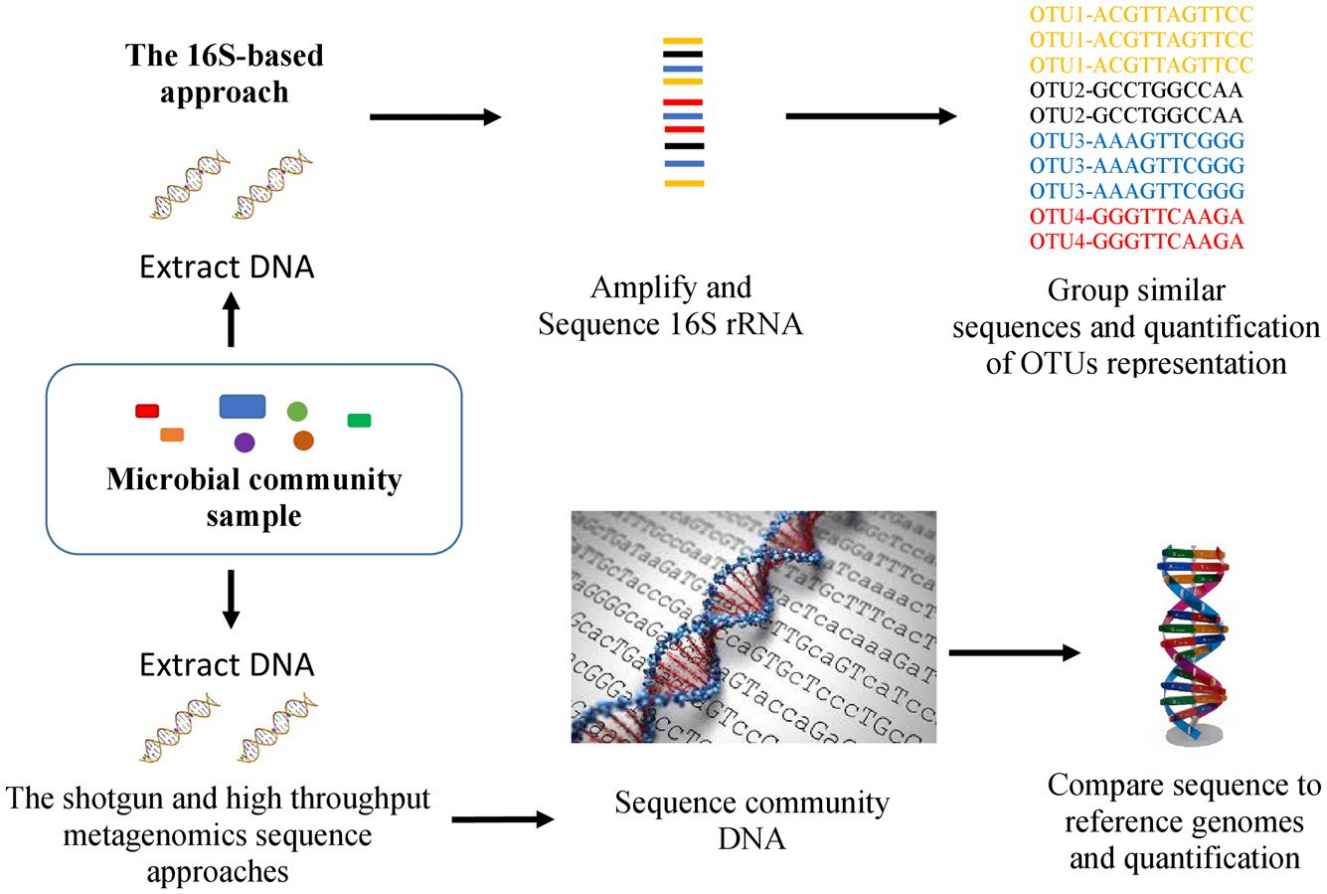
Shotgun and high‐throughput metagenomics, and 16S‐based sequence approaches for assessing microbial populations in a given biological sample

Using these molecular genetic sequence methods, it will be possible to identify and quantify the different microbiome species and families in a given biological sample. Figure 1 shows a diagram that describes the procedures the assessment of microbial populations in a given biological sample using the three methods including shotgun and high‐throughput metagenomics, and 16S‐based sequence approaches.

While environmental factors such as diet and lifestyle have been shown to influence the microbiome composition, the role of the host genetics remains unclear.^28^ The microbiome structural complexity, the challenge of multiple comparisons, and the large effect of the environment, probably result in variability and inconsistency between the results of recent studies.^28^ Therefore, the question of the extent to which human genetics shapes the microbiome composition remains open. In this review, we will present the recent studies and discuss their results in order to provide a current assessment of the role of host genetics in shaping the gut microbiome composition. Additionally, we will review the major insights from mouse studies and discuss how this model can contribute to future human microbiome research.

## 2 ASSESSMENT OF THE MICROBIOME HERITABILITY FROM TWIN STUDIES

**FIGURE 2:**
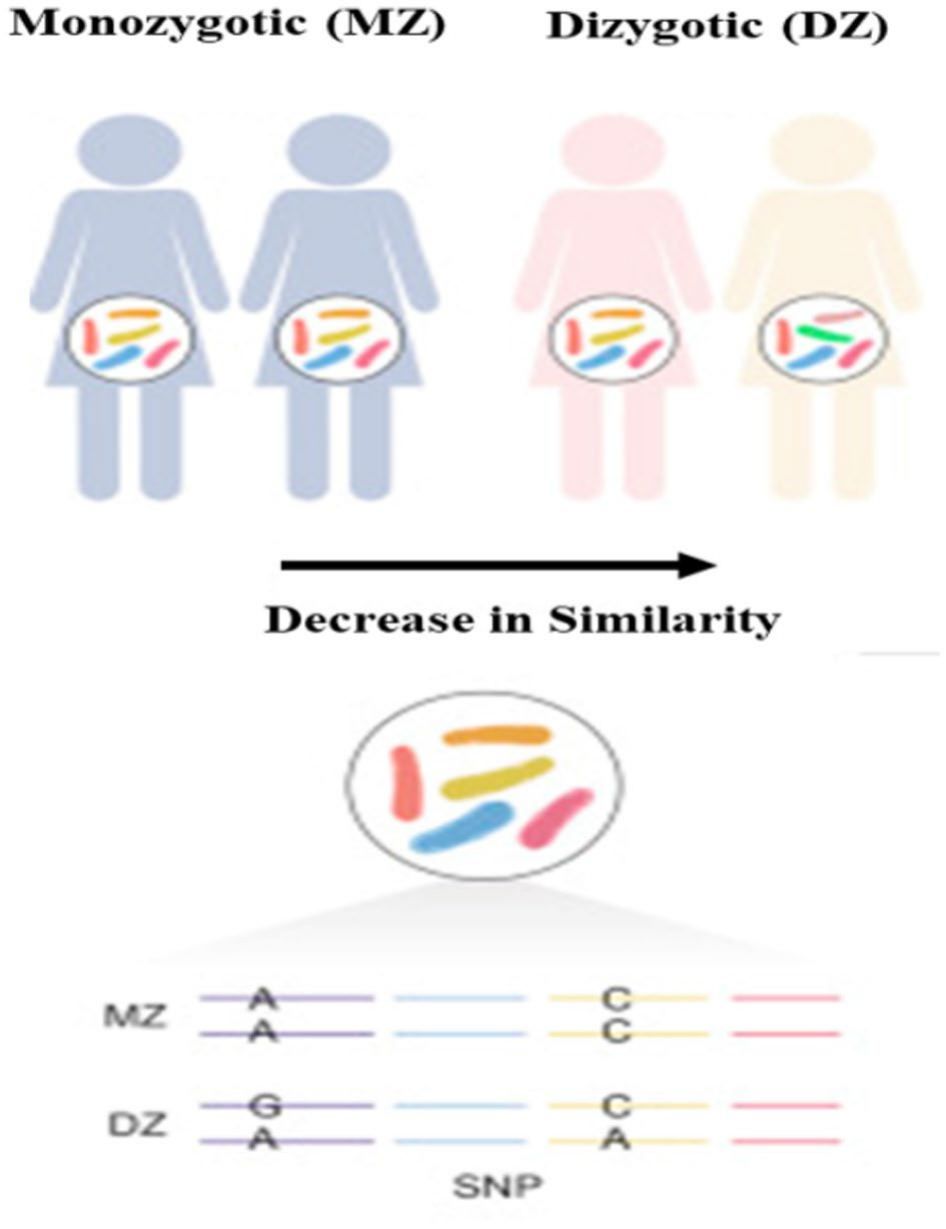
Microbial similarity in monozygotic twins, while genetic diversity is observed in dizygotic twins

The term “heritability” describes the inheritance extent of quantitative traits (eg, the abundance of gut bacteria), or in other words, the proportion of variation in a trait that can be explained by the host genetic.^29^ In order to investigate and assess the role of genetic background that determines the characteristics of the microbiome, environmental effects should be minimized as much as possible. One of the effective approaches is to compare the differences between groups of monozygotic (MZ) and dizygotic (DZ) twins.^28^, ^30^ Assuming that each type of twin experiences the same environmental conditions and that there is a difference in genetic similarity between MZ (100%) and DZ (~50%) twins, the heritability of traits can be estimated. Figure 2 represents a diagram shows the microbial similarity in monozygotic twins, while genetic diversity is observed in dizygotic twins.

Although twin studies are the basic method to investigate whether genetic variation in the host is related to genetic variation in the microbiome, they have not provided the same answers over the years.^8^, ^9^ Earlier studies reported that there was no significant difference in gut microbiome composition between MZ and DZ twins.^31^, ^32^ In contrast, recent studies have shown opposite results suggesting that the abundances of specific members of the intestinal microbiota are slightly influenced by host genetics.^8^, ^33^ While the first two studies^31^, ^32^ were based on relatively small cohorts (54 and 87 twin pairs, respectively), the current studies^8^, ^9^ significantly increased the sample size (416 and 1,126 twin pairs, respectively) and therefore were able to reveal the associations. By using 16S rRNA gene‐based analysis, they identified many heritable taxa.^34^, ^35^ The most heritable was the family Christensenellaceae (phylum Firmicutes), which was also the hub of a co‐occurrence network that includes other taxa with high heritability. Furthermore, this heritable taxon was enriched in individuals with low BMI, and adding it to an obese‐associated microbiome reduced weight gain in transplanted germ‐free mice. These observations indicate that heritable microbes can directly contribute to the host phenotype and that the microbial phenotype is additionally influenced by host genetics.^35^ In 2016, a follow‐up study that included both previous and new data were published.^9^ The expanded dataset resulted in heritability estimates with narrower confidence intervals and revealed additional heritable taxa. They discovered that heritable taxa are temporally stable over long periods, suggesting that their relative abundances are less affected by environmental factors. To further investigate the involvement of host genetics, another study on a subset of Twins‐UK participants (250 twins) was performed, using metagenomics shotgun sequencing for the analysis.^36^ They showed heritability not only for many microbial taxa but also for functional modules in the gut microbiome that can relate to the risk of complex diseases. When comparing to previous results that used 16S profiles, most of the microbiome heritability in this study were higher, suggesting that metagenomics analyses can provide greater resolution and power.^36^ However, as the twins began to live apart, the microbial similarity between them declined, indicating that the environment overshadows genetics in the gut microbiome design.^36^, ^37^ A recent twin study found associations between high heredity microbial taxa and visceral fat accumulation, indicating host genetics as a potential mediator between obese complex phenotype and gut microbiome composition.^38^

Contrary to the findings presented above, several non‐twin studies showed a significant bacterial similarity among genetically unrelated individuals who shared a household, but such similarity was not observed across family members that did not share a household.^39^, ^40^, ^41^ These results indicate that the gut microbiome is primarily shaped by environmental factors and that the effect of host genetics is apparently quite modest.

## 3 ASSOCIATIONS BETWEEN HOST GENETICS AND GUT MICROBIOME

One of the main approaches to investigate microbiome‐host genome associations is microbial genome‐wide association studies (mGWAS), which are cohort studies that combine human genotyping or whole‐genome sequencing with microbiome analysis (16S rRNA or metagenomics sequencing) from the same individual.^42^ Newer and cheaper technologies in recent years have enabled the discovery of several gut microbiomes associated with SNPs. These are related to the innate immune system or metabolism and are located near host genes associated with complex diseases.^9^, ^33^, ^43^, ^44^, ^45^, ^46^, ^47^, ^48^, ^49^ However, most of the associations were not statistically significant after multiple testing corrections and the reported variants were not repeated in different studies.^28^, ^40^ Therefore, these results are limited.

The great complexity of the microbiome structure and the strong environmental effects probably result in variability and inconsistency between the results of recent studies.^28^, ^37^ Furthermore, an analysis of 5 previous studies, which included 225 SNPs significantly associated with microbiome parameters, showed almost no overlap between the loci reported in different studies.^40^ However, the lactase gene (LCT) is an exception. In fact, the association between LCT locus and the relative abundance of the *Bifidobacterium* (*phylum* Actinobacteria) is the only consistent finding that has been validated in subsequent cohorts.^40^, ^42^, ^45^ The lactase enzyme is responsible for the breakdown of lactose and encoded by the LCT gene, whose variants are associated with lactase persistence and lactose tolerance in adults.^9^ *Bifidobacterium* is part of the gut microbiome composition that can utilize lactose, the milk sugar, as an energy source. Thus, its association with the LCT gene may be mediated through environmental factors like diet and lactose consumption that are also influenced by culture and geography.^9^, ^50^ The different genotypes of this SNP showed associations with different levels of the *Bifidobacterium*. To be more specific, the SNP that is typically associated with lactase nonpersistence was also associated with the elevated abundance of *Bifidobacterium*.^34^ When combining the dietary information of the subjects, the association was only in lactase non‐persistence individuals that consumed lactose.^45^ This finding can explain why the phenotype predicted by the genotype (lactose tolerance or intolerance) is not always accurate.^51^ In some cases, the microbiome has more influence than the genotype on the observed phenotype.^15^

Studies that used another approach, which defines the entire microbiome composition as one feature (β‐diversity) rather than treating taxa separately, managed to find several genetic associations for the overall microbial variation,^52^ for example, in VDR gene (vitamin D receptor).^37^, ^49^ In addition, it was argued that 42 SNPs can explain over 10% of the β‐diversity variance,^49^ but since further studies failed to replicate this, the association of any individual SNP with microbiome β‐diversity is very limited.^40^, ^53^ In contrast, when the associations with environmental factors related to diet and lifestyle were examined, they explain over 20% of the variance in microbiome β‐diversity and thereby indicate greater environmental influence than genetic influence.^34^, ^40^, ^53^, ^54^

## 4 MAJOR INSIGHTS FROM MOUSE STUDIES

Animal models, in which genetics and environmental factors are better controlled, can be used to further investigate the role of host genetics. Among all nonhuman models, the mouse has been the most common and productive model to investigate the gut microbiome to date.^55^, ^56^ Besides high genetic similarity, the mouse shows some similarity to humans at the microbial taxonomic level, thus considered a powerful model for evaluating host‐microbiota interactions applicable to humans.^55^, ^57^, ^58^ Many mouse studies have demonstrated a consistent and reproducible impact of host genetics on the gut microbiome composition.^57^, ^59^, ^60^, ^61^, ^62^ For example, the genetic background has shown to be a stronger determinant than gender on the mouse microbiome, since the similarity of microbiota composition within mouse lines was significantly higher than between different lines or within same‐sex groups.^62^ A recent analysis of F1 hybrids generated from a reciprocal cross between two inbred lines (BALB/c, C57BL/6J) showed a greater genetic effect on the microbial composition than maternal effect or continuous exposure to different microbiota of the other line.^61^ These results are compatible with previous studies,^63^, ^64^ thus reinforce the hypothesis about the existence of a line‐dependent bacterial signature that each line tends to maintain.^61^ In addition, several heritable microbial taxa such as *Lactobacillus johnsonii* were identified,^61^ in which their relative abundance was affected predominantly by host genetics.^60^ It was also discovered that host genetic distance is associated with the overall microbiome composition (β‐diversity).^65^ By using quantitative trait locus (QTL) mapping methods, studies have managed to find several genetic loci that influence specific microbial taxa or pathways, most related to the immune system and metabolism.^28^, ^59^, ^66^, ^67^, ^68^ A recent study found a significant overlap between genetic associations discovered in wild mice and humans, in particular genes related to the nervous system, the immune system, and obesity, suggesting a possible mammalian‐shared genetic mechanism that affects the gut microbiome composition.^65^

**FIGURE 3:**
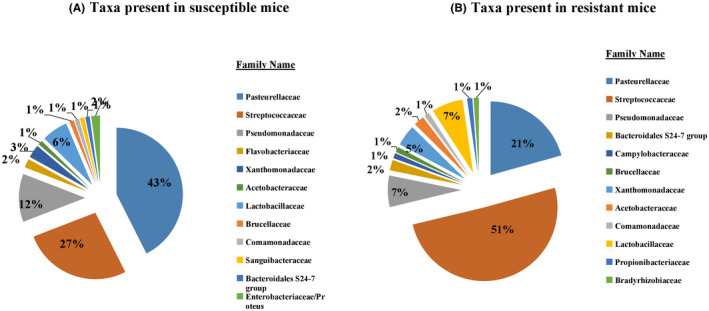
Percentage profiles of the top bacteria families that found in the normal flora in susceptible (3A) and resistant mouse populations (3B) to a specific environmental challenge. While 43% of the Pasteurellaceae taxa was found in the susceptible mouse population (3A), only 21% of this taxa was found in resistant mouse samples (3B) to the same environmental challenge. Streptococcaeae taxa was found 27% and 51% in susceptible (3A) and resistant (3B) mouse populations, respectively, to the same environmental challenge (Unpublished data)

Figure 3 shows an example of percentage profiles of the top bacteria families that are found in the normal flora in susceptible (3A) and resistant mouse populations (3B) to a specific environmental challenge. As shown in Figure 3A, 43% of the Pasteurellaceae taxa was found in the resistant mouse population, while only 21% of this taxa was found in the susceptible mouse population. Interestingly, as shown in Figure 3B, Streptococcaeae taxa were found to be 27% and 51% in susceptible and resistant mouse populations, respectively (Unpublished personal data). Interestingly, B Streptococcaeae taxa was found to be 27% and 51% in susceptible and resistant mouse populations, respectively (Unpublished personal data).

## 5 MICROBIOME HERITABILITY

The heritability of the trait in the study population tells us the correlation between the observed phenotype of an individual in that population and the true genetic value of the individual. Since the heritability is usually less than 1.0, this means that an individual with a given observed phenotype can have a true genetic value that varies more or less widely about that phenotype. Calculating and estimating the hereditability rate of the gut microbiome profiles are important in determining the level of the host genetic effect on the microbiome patterns. It is known that as the sample size is increased, the estimated heritability becomes more powerful and accurate.^9^ Nevertheless, in previous studies, the estimated heritability of gut microbiome profiles was found to be relatively low compared to other various complex traits tested in the same research populations.^69^, ^70^, ^71^ While the estimated heritability of the microbial taxa in some of the reported studies was ranged between 0.10 and 0.30, the estimated heritability of other assessed phenotypic traits was much higher. These findings supported the previous statements, that several gut bacteria are heritable; however, the overall heritability tends to be smaller than it was initially estimated and expected.^37^, ^40^

## 6 DISCUSSION

The human gut microbiome is increasingly recognized as a key player in maintaining our health. Multiple factors shape its composition; however, the extent to which host genetics shape the microbiome composition remains controversial. One of the classic methods to investigate heritability is to examine differences between MZ and DZ twins.^28^ Previous twin studies have shown that the overall microbiome composition^8^ and many individual taxa are heritable.^9^, ^36^, ^38^, ^72^ Although the studies usually identified the same heritable taxa, the degree of heredity was not the same, probably due to differences in sample size and method of analysis.^36^, ^40^ These research characteristics are very important for the significance of heritability results, especially in twin studies on the microbiome. Therefore, larger scale studies in the future may yield additional results that will clarify the extent of the microbiome heritability.

Although we can conclude from the twin studies presented above that host genetics is an integral factor in shaping, at least part, of the gut microbiome composition, it should be kept in mind that non‐twin studies provide evidence for the dominant role of the environment in shaping the gut microbiome.^39^, ^40^, ^41^ It should also be noted that compared to other complex traits tested in twin studies, the heritability of the microbiome components was relatively low.^69^, ^70^, ^71^ While these findings do not contradict the heritability revealed in the twin studies, they do suggest that the existing genetic influence is relatively weak.

Finding associations between genetics and complex traits is a challenging task and when it comes to the gut microbiome, the challenge is sometimes even harder. In recent years, many studies have been published, mainly due to the great technical progress and the decrease in the cost of sequencing.^73^ Although much effort has been invested, it is still difficult to deduce a clear trend regarding the role of host genetics in the gut microbiome. This difficulty is caused by the inconsistency and unevenness between the studies' results. Most of the detected SNPs were not repeated in subsequent studies and there was almost no overlap between their reported loci.^28^, ^40^ This suggests that most of the SNP‐bacteria associations are either weak or population dependent.^40^ In addition, a growing number of studies show that environmental factors can explain a greater proportion of the microbiome variability compared to the host genetics.^9^, ^40^, ^53^, ^54^ Therefore, the hypothesis that the gut microbiome is predominantly shaped by environmental factors is reinforced.

An exceptional example is a significant association between the LCT gene and the relative abundance of *Bifidobacterium*, which has been consistently repeated across studies.^40^, ^42^, ^45^ However, this association may be mediated by lactose consumption.^9^, ^45^ This important finding offers a potential mechanism of gene‐environment interactions in regulating the gut microbiome variability. It also suggests that more associations may be revealed by looking at the combined effect of genetics and the environment rather than exploring their effects separately.

Despite the inconsistencies in the results, it is important to keep in mind that associations identified in GWAS can be difficult to replicate in other studies because of small effect sizes of single variants, variability between study participants in terms of geography and genetic background, and differences in study design.^33^ As presented in twins, larger scale future studies may reveal additional microbiota‐associated SNPs but are unlikely to change the overall conclusion that the gut microbiome is mostly shaped by non‐genetic factors.^40^

In human studies, large cohort sizes are necessary to uncover the genetic effects on the microbiome since the wide environmental variation among the participants cannot be completely controlled.^15^ However, mouse studies can minimize this variation by exposing all mice to the same environmental conditions.^58^ Mouse studies, in which the confounding factors are better controlled, suggesting a greater role of host genetics in shaping the gut microbiome than the role presented in humans. Furthermore, from the human and mouse studies discussed in this work, it can be said in general that results obtained in mice were more consistent and replicable, thus presenting a more reliable trend regarding the host genetic background importance. However, these findings raise the question of how representative the mouse model is for human microbiome research. While previous studies have argued that there is a high similarity between mouse and human in the gut microbiome composition, recent analyzes challenge this assumption and suggest far less similarity. Therefore, conclusions must be drawn with caution since the results in mice referring to the intestinal microbiome do not always applicable to humans.^56^, ^74^ Nevertheless, a comparison of the associations identified by mGWAS in both humans and wild mice yielded surprising findings.^65^ First, the number of genes that overlapped was significantly larger than it was randomly expected, suggesting a possible conserved set of microbiome‐associated genes across various mammals. Second, since the overlap host genes were related to the immune system, brain, and obesity, they can serve as strong potential candidates for future research.^65^ Despite the dissimilarity of the microbiome system between mice and humans, the mouse model presents important advantages and can provide some insights and future directions for human research.

In conclusion, a number of previous studies have shown that the host genetic background is a fundamental component in determining the composition of the gut microbiome and profile. Furthermore, the composition of the gut microbiota is also known to be modulated by multiple factors, including the age, sex, and diet of the host.^75^ Increasing evidence from mouse models has suggested that host genetics also contribute to shaping gut microbiota.^44^, ^59^ Recently, Goodrich et al^35^ found that a number of gut microbial taxa, including Christensenellaceae, were heritable in humans, which strongly suggests that the human gut microbiota can be influenced by host genetics. At the level of specific locus, several studies have demonstrated that variations in several host genes, such as nucleotide‐binding oligomerization domain 2 and fucosyltransferase 2, may contribute to alterations in gut microbial community structure, and consequently, affect Crohn's disease (CD) susceptibility in humans.^76^ Therefore, it can be hypothesized that metabolic syndrome (MetS), gut microbial composition, and host genotype are inter‐related. However, to our knowledge, no studies to date have investigated which specific gut microbes are responsible for MetS and whether such microbes are related to single nucleotide polymorphisms (SNPs), in particular host genes.

Interestingly, it appears that the effect of the host genetic structure is strong at young ages and seems to be reduced over time, while the environmental impact is getting stronger at older ages, and interperson microbiome variability becomes to be more influenced with environmental factors related to diet, drugs, and anthropometric measurements. Although it is now widely accepted that the gut microbiota plays a crucial role in host metabolism, the current knowledge on the effect of host genetics on specific gut microbes related to MetS status remains limited. In addition, accumulated data have suggested that dysbiosis of the gut microbiota is closely related to the development of obesity and, subsequently, other hallmarks of MetS. These data imply a critical role of the gut microbiota in the development of MetS in humans, and very few studies have investigated the gut microbiota of individuals in relation to MetS. Unfortunately, as of today, there are a limited number of reports that show the association of host genetic factors with hereditary of gut microbiome profiles, affected by diet, metabolism, and obesity phenotypes. Evaluating the full role of host genetics on its various aspects is challenging and inaccurate since the current research methods are probably not strong enough to detect all genetic effects. Continued microbiome research is essential to understand the comprehensive genetic potential of the host. In addition, comparing results from mice and humans can provide potential candidate genes and guide the focus of future human research.

## CONFLICT OF INTEREST

None.
